# The Noninvasive Treatment for Sentinel Lymph Node Metastasis by Photodynamic Therapy Using Phospholipid Polymer as a Nanotransporter of Verteporfin

**DOI:** 10.1155/2017/7412865

**Published:** 2017-04-03

**Authors:** Kyosuke Shimada, Sachiko Matsuda, Hiromitsu Jinno, Noriaki Kameyama, Tomohiro Konno, Tsunenori Arai, Kazuhiko Ishihara, Yuko Kitagawa

**Affiliations:** ^1^Department of Breast Surgery, Kawasaki Municipal Ida Hospital, 2-27-1 Ida, Nakahara, Kawasaki, Kanagawa 211-0035, Japan; ^2^Department of Surgery, Keio University School of Medicine, 35 Shinanomachi, Shinjuku, Tokyo 160-8582, Japan; ^3^Endowed Chair in Cancer Research, Keio University School of Medicine, 35 Shinanomachi, Shinjuku, Tokyo 160-8582, Japan; ^4^Department of Surgery, Teikyo University School of Medicine, 2-11-1, Kaga, Itabashi, Tokyo 173-8606, Japan; ^5^Department of Surgery, Tachikawa Hospital, 4-2-22, Niishikicho, Tachikawa, Tokyo 190-8531, Japan; ^6^Department of Bioengineering, School of Engineering, The University of Tokyo, 7-3-1 Hongo, Bunkyo, Tokyo 113-8656, Japan; ^7^Faculty of Science and Technology, Keio University, 3-14-1 Hiyoshi, Kohoku, Yokohama, Kanagawa 223-8522, Japan; ^8^Department of Materials Engineering, School of Engineering, The University of Tokyo, 7-3-1 Hongo, Bunkyo, Tokyo 113-8656, Japan

## Abstract

*Aim*. The usefulness of photodynamic therapy (PDT) for treating sentinel lymph node (SLN) metastasis was evaluated.* Materials and Methods*. Verteporfin, a hydrophobic photosensitizer, forms a soluble aggregate with poly(2-methacryloyloxyethyl phosphorylcholine-*co*-*n*-butyl methacrylate) (PMB). The concentrations of verteporfin were determined by measuring the fluorescence emitted at 700 nm. Seven days after the inoculation of A431 cells at the forearm of BALB/c nude mice, PMB-verteporfin was injected at dorsum manus and 75 J of light energy was delivered for 1 minute. Fifty-three mice were randomly assigned to the combination of PMB-verteporfin injection and light exposure, light exposure alone, PMB-verteporfin injection alone, and no treatment groups. Ten days after PDT, brachial lymph nodes, which were considered as SLNs, were harvested and evaluated.* Results*. The concentration of verteporfin in SLN was significantly higher than other organs. The combination of PMB-verteporfin injection and light exposure group significantly reduced the SLN metastasis (13%) comparing with no treatment group (52%), light exposure alone group (57%), and PMB-verteporfin injection alone group (46%).* Conclusions*. These data suggested that PDT using PMB as a nanotransporter of verteporfin could be a minimally invasive treatment of SLN metastasis in breast cancer and represent a potential alternative procedure to SLNB.

## 1. Introduction

Axillary lymph node dissection (ALND) has been integral part of breast cancer surgery since the description of the radical mastectomy [1]. The ALND can achieve good local disease control and the meta-analysis concluded that local control of breast cancer is associated with improved disease-specific survival [2]. The management of the axilla, however, has changed radically with the introduction of the sentinel lymph node biopsy (SLNB) in the early 1990s [3]. The first lymph node (LN) that receives drainage from a primary tumor is defined as sentinel lymph node (SLN) and when metastasis is not found in an SLN, it almost certainly will not be present in more distal LN. In this concept, the primary benefit of SLN mapping and biopsy is that it enables surgeons to avoid nontherapeutic ALND. Veronesi et al. found that SLNB is a safe and accurate method of screening the axillary nodes for metastasis in women with a small breast cancer by the randomized trial [4].

The SLNB has become a gold standard procedure for axillary lymph node evaluation in clinically node-negative patients, and emerging data show that the survival benefits of the ALND may not be greater than the SLNB alone in patients with up to 2 positive SLNs [5–7]. In other words, most of breast cancer patients do not need the ALND and could be treated with the SLNB alone.

Although the SLNB is much less invasive comparing with the ALND, it is still associated with complications such as lymphedema, numbness, and pain [8–10]. Moreover, blue dye and radioactive tracer, which were used to detect SLNs, might cause some problems, such as anaphylaxis shock and exposure to radiation. Therefore, less invasive treatment against SLN metastasis needs to be developed.

A photodynamic therapy (PDT) involves the systemic or local administration of photosensitizer followed by its subsequent activation by broadband red light. In the presence of oxygen, the activated photosensitizer can generate reactive oxygen species that cause cell damage and ultimately cell death [11]. Verteporfin is a hydrophobic polyporphyrin oligomer with two structural isomers, a short photosensitivity period [12], and maximum absorption at 689 nm. The verteporfin has been approved for PDT of abnormal blood vessels in the eye, the wet form of macular degeneration. Although several studies have evaluated its therapeutic potential use in cancers [13–18], most of these studies have been performed in vitro using cell lines, and photosensitizers often show poor specificity for tumor tissue, limiting their application in cancer treatment.

**Figure 1:**
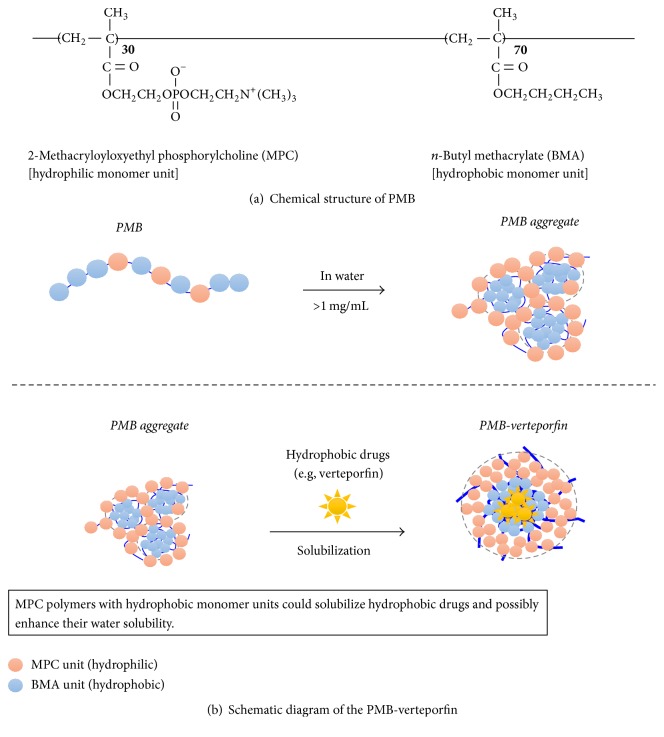


A 2-methacryloyloxyethyl phosphorylcholine (MPC) polymer has the same polar group (phosphorylcholine group) of phospholipids constructed as cell membranes and possesses excellent biocompatibility, that is, reduction of protein absorption and inhibition of platelet adhesion at the surface of the MPC polymer [19, 20]. Thus, the MPC polymers have been utilized as surface modifiers in many medical devices in order to improve biocompatibility. By changing the molecular design of the MPC polymers, we have obtained water-soluble and amphiphilic MPC polymers. For example, one of the MPC polymers, poly(MPC-*co-n*-butyl methacrylate) (PMB) with 30 unit% of MPC units and molecular-weight below 5.0 × 10^4^ can be dissolved in an aqueous medium and form stable polymer aggregates [20]. The hydrophobic part of the polymer provides hydrophobic domain in the polymer aggregate and could solubilize hydrophobic reagents and enhance their water solubility [21] (Figure 1). It is already confirmed that when an aqueous solution of PMB injection is carried out into rabbit vain directly, no significant effects on blood functions can be observed [21]. Therefore, the possibilities of the PMB being used as a transporter for verteporfin in vivo, which is very poorly soluble in aqueous media, were explored.

In this study, the efficacy of PDT using water-soluble and amphiphilic PMB as a nanotransporter of verteporfin for the noninvasive treatment of SLN metastasis was evaluated.

## 2. Materials and Methods

### 2.1. Cell Lines

Epidermoid carcinoma A431 cells (ATCC-No. CRL-1555) were obtained from the American Type Culture Collection (Manassas, VA, USA). The A431 cell line with stable expression of green fluorescence protein (GFP) (A431-GFP cells) was obtained by transfection of pEGFP-N1 (Promega, Madison, WI, USA) followed by G418 selection. The cells were maintained in DMEM supplemented with 10% heat-inactivated foetal bovine serum (Gibco, Grand Island, NY, USA) in a humidified atmosphere of air containing 5.0% CO_2_ at 37°C.

### 2.2. Animals

All animal experiments were conducted according to Keio University's institutional guidelines for the care and use of laboratory animals in research. BALB/c nude mice were purchased from Oriental Yeast Co., Ltd. (Tokyo, Japan). They were maintained under specific pathogen-free conditions in the Keio University Experimental Animal Center on a standard laboratory chow diet and had access to tap water ad libitum. Six-week-old female mice weighing 15 to 20 g were used in experiments.

### 2.3. Establishment of Murine SLN Metastatic Model

**Figure 2:**
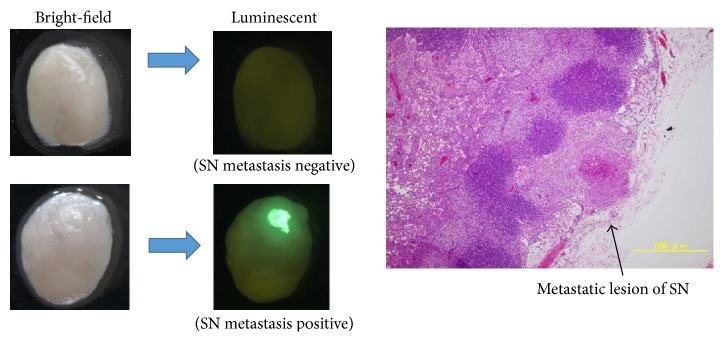
Stereoscopic fluorescence microscope images of metastatic sentinel lymph nodes.

Murine SLN metastatic model was developed by subcutaneous injection of 5 × 10^5^ A431-GFP cells/50 *μ*L at forearm of BALB/c nude mice. The brachial lymph nodes, which were considered as SLNs, were harvested after 7 days and examined by stereoscopic fluorescence microscope (Figure 2).

### 2.4. Preparation of PMB-Verteporfin

PMB was synthesized and purified as previously described. The composition of the MPC units and BMA units was 30 mol% and 70 mol%, respectively. Verteporfin was dissolved in dichloromethane at concentration of 100 mg/mL. In the meanwhile, the PMB was dissolved at concentration of 50 mg/mL in PBS. Then, 200 *μ*L of verteporfin solution was added to 5.0 mL of the PMB solution dropwise on ice. The mixture was sonicated for 30 min with a sonicator, Branson Sonifier 450 (Branson, Danbury, CT, USA), on ice and stirred on a magnetic stirrer for 1.0 h at room temperature in order to evaporate dichloromethane. Finally, aqueous solution of verteporfin in PMB aggregate (PMB-verteporfin) was obtained.

### 2.5. Measurement of Diameter of PMB Aggregate and PMB-Verteporfin

The diameter of each component was measured using a particle size analyser, Zetasizer nano (Malvern Instruments, Malvern, UK).

### 2.6. Measurement of Verteporfin Concentration In Vivo

PMB-verteporfin (4 mg/mL) was administered as single bolus injections at each dorsum manus of 12 mice, to give a dose of 0.2 mg/body. One hour later, organ samples including SLN, lung, liver, kidney, and brachial skin were harvested from each mice, weighed, and lyophilized. N,N-dimethylformamide was added to each freeze-dried samples, which were then homogenized using a MagNA Lyser (Roche, Mannheim, Germany) at 6,500 rpm for 30 sec and centrifuged to extract verteporfin. The concentration of verteporfin was calculated from the fluorescence emitted at 700 nm (excitation at 430 nm) using the microplate reader, Synergy 4 Multimode (Bio Tek, Vermont, USA).

### 2.7. Evaluation of the Inhibitory Effect of PDT against SLN Metastasis

**Figure 3:**
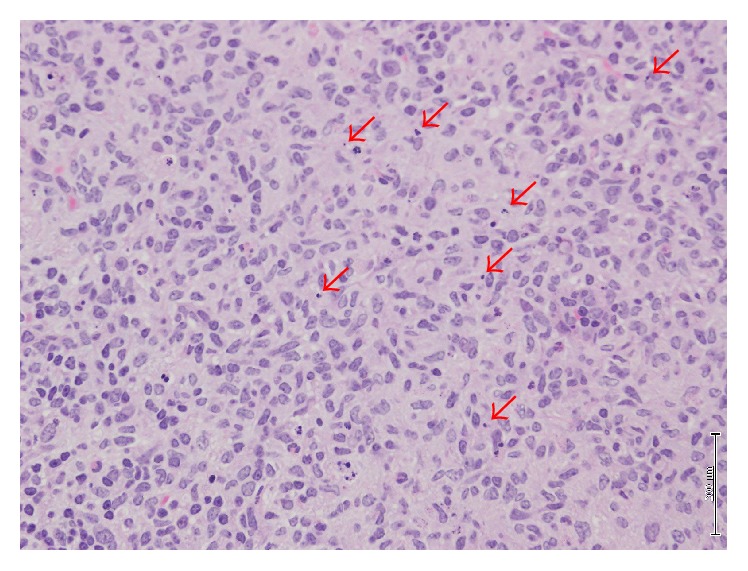
Nuclear disruption in SLN after PDT.

Fifty-three mice with subcutaneous injection of A431-GFP cells at the forearm were divided into 4 treatment arms including the combination of PMB-verteporfin injection and light exposure, light exposure alone, the PMB-verteporfin injection alone, and no treatment. The PMB-verteporfin was subcutaneously injected at dorsum manus 7 days after inoculation of A431-GFP cells. One hour later, mice were exposed to a diode laser light (at 640 nm) using an Optical Fuel laser (Sony, Tokyo, Japan). Q-band excitation was established at this wavelength. The light dose was 75 J/cm^2^ for a total treatment time of 1 minute and irradiance ranged from 0.18 to 0.76 W/cm^2^. During irradiation the temperature was kept at 20°C. After 10 days from PDT, the SLNs were harvested and evaluated by stereoscopic fluorescence microscope. The microscopic image of SLN treated with PDT revealed a small amount of nuclear disruption (Figure 3).

### 2.8. Statistical Analysis

The concentration of verteporfin was given as means ± standard deviation. The SPSS PASW Statistics 18 (IBM Corporation, Armonk, NY, USA) was used for analyzing the difference of concentration using unpaired Student's *t*-test. The inhibitory effect on SLN metastasis was analysed in Mann-Whitney *U* test using SPSS PASW Statistics 18. The *p* value of <0.05 was defined as being statistically significant.

## 3. Results

### 3.1. Physical Characterization of PMB-Verteporfin

**Figure 4:**
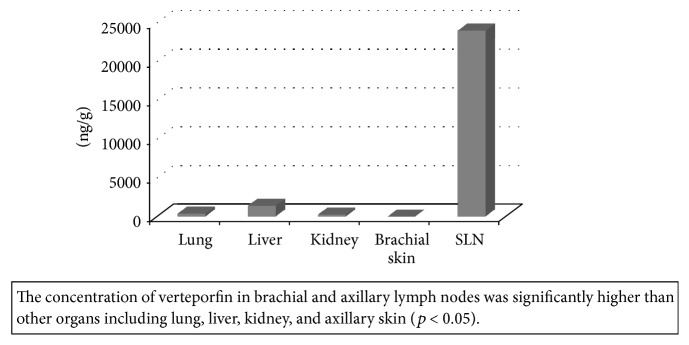
Amount of verteporfin distributed in various tissues.

The verteporfin could be solubilized in the PMB solution well and clear solution was obtained. That is, a stable conjugate was formed. The diameters of PMB aggregate without and with verteporfin were 197 ± 9 nm and 178 ± 28 nm, respectively. Concentration of verteporfin was 23.9 ± 3.8 *μ*g/g tissue in SLNs. Concentrations of verteporfin in lung, liver, kidney, and brachial skin of mice were 0.38 ± 0.38 *μ*g/g, 1.38 ± 0.93 *μ*g/g, 0.26 ± 0.06 *μ*g/g, and not detected, respectively (Figure 4).

The concentration of verteporfin in SLNs was significantly higher than other organs (*p* < 0.05). The injection site of PMB-verteporfin did not show any damage including inflammation or ulceration.

### 3.2. Evaluation of the Inhibitory Effect of PDT against SLN Metastasis

**Figure 5:**
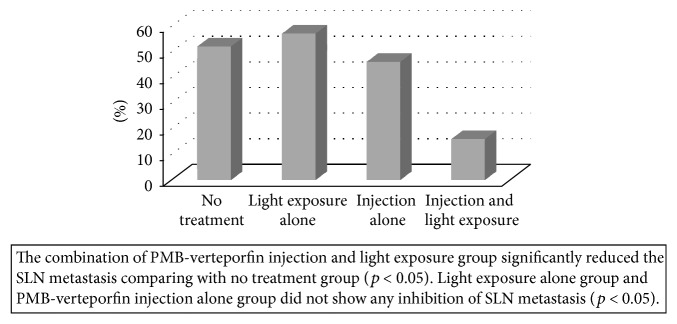
Effect of PDT on SLN metastasis.

The combination of the PMB-verteporfin injection and light exposure group significantly reduced the SLN metastasis comparing with no treatment group (13% versus 52%, *p* < 0.05) (Figure 5). Inhibition of SLN metastasis was not found in light exposure alone group (57%) and PMB-verteporfin injection alone group (46%). Inhibitory effect of the combination group was significantly higher than light exposure alone group and injection alone group (*p* < 0.05).

## 4. Discussion

In this study, the PMB efficiently delivered verteporfin to the SLN via subcutaneous injection and the PDT using the PMB as a nanotransporter of verteporfin revealed inhibitory effect against SLN metastasis.

The PDT is still not widely used for cancer treatment because of the limited specificity of the photosensitizers for cancerous tissue. In other words, healthy noncancerous tissue may also be damaged by photosensitizers following irradiation. One of the major complications of the PDT was cutaneous phototoxicity because photosensitizers might accumulate in normal tissue such as skin. After the infusion of the photosensitizers, patients are required to avoid direct sunlight for a period of 7–10 days on average [22, 23]. Therefore, improvement of delivery of photosensitizer to tumor is necessary for this approach to become widely used in the treatment against cancer. In this study, the concentration of verteporfin in organs except the SLN was extremely low. Verteporfin, especially, was not detected in brachial skin. This specific distribution of verteporfin could prevent several adverse events including cutaneous phototoxicity.

Several studies have been conducted on the correlation between the identification rate of the SLN and the size of radioisotope colloids and found that small-sized (200–400 nm) colloids were superior to regular-sized (400–1000 nm) colloids both in the detection rate by lymphoscintigram and the intraoperative identification rate of the SLNs [3, 24]. The diameter of the PMB-verteporfin was around 200 nm and this size was effective to be taken up by lymphatic channels and accumulate in lymph nodes. As a result, accumulation of the PMB-verteporfin in the other organs including brachial skin was reduced. Moreover, the PMB-verteporfin was administered not systemically but locally in our study and this injection route might contribute to the specific delivery of verteporfin to SLNs.

Among several photosensitizers, the verteporfin was picked up in our study because its activation wavelength (689 nm) is longer than other systemic PSs. Light with long wavelength can penetrate tissue deeply. Light with longer wavelength is necessary to reach SLN and activate PSs [12]. The treatable size of metastasis by PDT is still to be elucidated. Our previous study using murine model showed that PDT decreased the tumor size from almost 2,000 mm^3^ to 150 mm^3^ [11]. Moreover, phase 2 study of PDT against skin cancers revealed that the rate of histopathologic response, defined by absence of tumor on biopsy specimen, was 73% in tumor 1-2 cm in size [25].

Another advantage of the PDT might be repeated treatment. Although radiotherapy (RT) also could be useful to eradicate microscopic disease in axillary lymph nodes, RT usually could be given to patients only once. However, the PDT could be given to patients several times because of favourable toxicity profile.

Axillary lymph node status has traditionally been a guide to decide adjuvant treatment. However, current guidelines support differences in adjuvant systemic treatment based on the intrinsic subtype and multigene assay rather than the number of positive lymph nodes [26]. According to IBCSG23-01 study, which compared axillary dissection or not in breast cancer patients with micrometastatic sentinel lymph nodes, two groups did not differ in terms of proportions receiving adjuvant chemotherapy, indicating that axillary lymph node status had almost no influence on the decision of adjuvant treatment [27]. Results from AMAROS study comparing axillary dissection with radiotherapy in patients with positive sentinel node also showed that axillary dissection had no influence on the administration of adjuvant treatment [28].

The results of this study should be interpreted with caution. First, only one cancer cell line was used. Further studies using other cell lines would be valuable to confirm these findings. Second, fixed doses of verteporfin and light exposure were used. Although the doses used achieved good antitumor effects, dose-response studies would help to determine the optimum doses of both verteporfin and light exposure. Third, the lymph node metastasis rate of the murine SLN metastasis model in this study was 60%. Stable metastasis model with higher rate was necessary to validate the usefulness of PDT against SLN metastasis models.

Taken together, these results indicate that PDT using PMB as a nanotransporter of hydrophobic verteporfin might be noninvasive treatment against the SLN metastasis. As the roll of the ALND for primary breast cancer has been considered to diminish over recent years, patients with up to 2 SLNs involvement could be treated with the SLNB alone. Although the SLNB is much less invasive procedure comparing with the ALND, it is still associated with complications such as lymph edema, numbness, and pain.

The PDT using PMB-verteporfin could avoid the SLNB in most of clinically node-negative breast cancer patients.
